# Towards an All-Solid-State Electrochromic Device: A Review of Solid-State Electrolytes and the Way Forward

**DOI:** 10.3390/polym14122458

**Published:** 2022-06-16

**Authors:** Benedict Wen-Cheun Au, Kah-Yoong Chan

**Affiliations:** Centre for Advanced Devices and Systems, Faculty of Engineering, Multimedia University, Persiaran Multimedia, Cyberjaya 63100, Selangor, Malaysia; benedictau@gmail.com

**Keywords:** electrochromic, electrochromic device, solid-state electrolyte

## Abstract

In order to curb high electricity usage, especially in commercial buildings, smart windows, also known as “switchable” or “smart” glasses, have attracted a significant amount of attention in an effort to achieve energy savings in eco-friendly buildings and transportation systems. Smart windows save energy by regulating the input of solar heat and light and hence cutting down air-conditioning expenses, while maintaining indoor comfort. This is achieved by electrochromism, which is defined as the reversible colour change in electrochromic (EC) materials from transparent to dark blue and vice versa under a small applied voltage. Recent state-of-the-art electrochromic devices (ECD) adopt liquid-based electrolytes as the main source of energy for basic operations. While this has resulted in much success in ECDs as reported in past studies, there remain several drawbacks to this aspect, such as liquid electrolyte leakage and evaporation, not to mention safety concerns related to the harmful nature of electrolyte materials. This paper aims to review the recent advances in various solid electrolytes that are potential solutions to the mentioned problems.

## 1. Introduction

**Figure 1:**
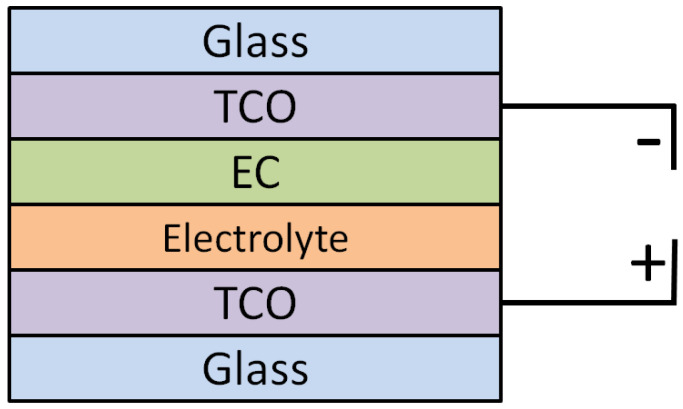
Device structure of a conventional ECD.

Smart windows revolve around the principle of electrochromism. This is the phenomena of inducing a reversible optical change (colouring or bleaching) in EC materials by applying a small electric field [1]. To explain further, the colouring and the bleaching of an EC material occurs because of insertion and extraction of ions, which is controlled by the applied voltage between transparent conducting oxide (TCO) layers [2]. Since the discovery of electrochromism by Deb in 1973, substantial efforts have been made to study EC materials due to its ability to regulate light in the visible region and produce energy savings in buildings for smart windows [3], displays [4] and automotive rear-view mirrors [5]. During the last several decades, many transition metal oxide materials have been examined for their EC properties, namely tungsten trioxide (WO_3_), titanium dioxide (TiO_2_), vanadium (V) oxide (V_2_O_5_), nickel oxide (NiO) and iridium oxide (IrO_2_). Among these materials, WO_3_ is one of the most studied EC materials due to its high colouration efficiency (CE), good reversibility ratio and fast colouration-bleaching speed [6]. In general, the EC phenomenon of WO_3_ is due to the formation of tungsten bronze (M_x_WO_3_) according to the electrochemical reaction below [7]:

WO_3_ (colourless) + xM^+^ + xe^−^ ←→ M_x_WO_3_ (dark blue) (1)

where M = H^+^, Li^+^, Na^+^ or K^+^ ions and x is ion concentration. Double injection occurs when M ions and electrons move into the EC layer, leading to the formation of dark blue film [8]. When the polarity of the applied voltage is reversed, the M ions and electrons are ejected out of the system, leading to a bleached state [9]. The fundamental structure of ECD is shown in Figure 1 below. It encapsulates two layers that are sealed between two transparent conductive oxide (TCO) coated glasses. These two layers are the EC and electrolyte layers [10]. Furthermore, each individual layer plays an important role in terms of device operation. TCO-coated glasses act as conducting layers, EC is the colour changing layer and the electrolyte layer provides the necessary ions for device operation [11]. 

In conventional ECDs, liquid polymer electrolytes (LPEs) are commonly adopted due to their high conductivity and ease of fabrication. These electrolytes are usually a combination of polymer plasticisers, which facilitate the reduction of the crystalline nature of the polymer matrix and also increase the polymer segmental mobility [12] and lithium salts, where its Li^+^ ions are incorporated into the EC layer, thereby colouring the ECD. Some examples of common polymer plasticisers are propylene carbonate, polyethylene glycol and polyethylene oxide. On the other hand, some examples of common lithium salts are lithium perchlorate (LiClO_4_), lithium iodide (LiI), lithium bis(trifluoromethanesulfonyl)imide (LiTFSI) and lithium hexafluorophosphate (LiPF_6_). Moreover, various combinations of polymer electrolytes can be fabricated to achieve high ionic conductivity [13]. 

Despite LPEs being advantageous in ECD applications, electrolyte leaking and evaporation, the formation of air bubbles and safety concerns related to the harmful nature of the organic solvent remain a challenge that may hinder their industrialisation for large area deployment [14]. To address the mentioned challenges, many researchers have attempted using various solid-state electrolytes (SSE) as an alternative to the existing liquid electrolyte technology. Table 1 presents a list of SSE candidates for application in ECDs. Poly (methyl methacrylate) (PMMA) is a thermoplastic material that has been widely studied for solid polymer electrolyte (SPE). It possesses high light transmittance and better impact resistance compared to glass [15]. Additionally, PMMA is commonly used in plasticised polymer electrolytes as a host polymer because it provides high ionic conductivity and chemical stability, with suitable inorganic lithium salts [16]. Nonetheless, given the advantages of PMMA in the SPE mixture, a minor compromise is the poor mechanical properties at high PMMA concentration, which greatly increase the viscosity of the electrolyte [17]. An alternative host polymer is gelatine. Gelatine is animal protein produced by partial hydrolysis of collagen, which can be found in animal skin, bone and connective tissue [18]. It is a biopolymer added to SPEs due to its high transparency, tackiness and adhesive properties [19]. Moreover, gelatine-based SPEs have welcomed promising outcomes from researchers [20,21,22,23]. Nafion membranes are among the alternatives widely used for proton exchange membrane fuel cells and water electrolysers [24]. These are ionic polymers made up of polyfluorocarbon backbone, with a high concentration of pendant sulfonic acid groups, which facilitates proton transfer between conducting electrodes. Over the years, Nafion has attracted interest in the field of EC owing to its good performance in fuel cells [25]. In terms of metal oxide materials, tantalum pentoxide (Ta_2_O_5_) is a promising substitution due to its high transparency, low leakage current and ability to act as a proton reservoir [26]. Various methods are used for the fabrication of Ta_2_O_5_, namely spin-coating, sputtering and pulsed-laser deposition [27]. Furthermore, significant studies on the use of Ta_2_O_5_ as SPEs have shown promising results in ECD applications [28,29,30]. 

In this work, a thorough review will be carried out of the aforementioned SSEs in terms of latest developments; in particular, fabrication methods, EC behaviours and distinctive features are highlighted and elaborated. This will enlighten the research community working on SSEs for ECD applications. 

### 1.1. Key Materials in Electrochromic Devices: PMMA-Based SPE ECD

**Figure 2:**
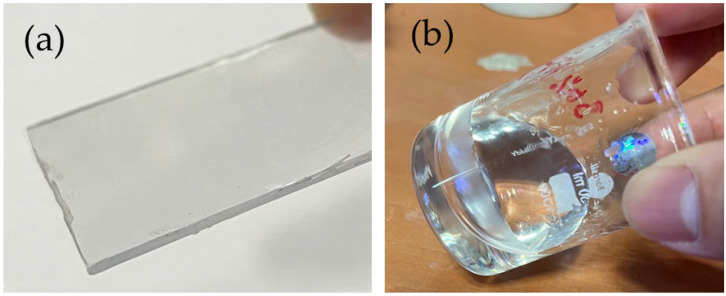
(**a**) Transparent PMMA acrylic sheet and (**b**) PMMA solid electrolyte.

As discussed earlier, PMMA is a thermoplastic material with a high level of transmittance in visible light and more impact resistant than glass. However, it comes with a minor compromise in terms of mechanical properties, where viscosity increases greatly at high concentrations, as shown in Figure 2b. In the last few decades, PMMA has been frequently studied as a host polymer in EC applications due to its high ionic conductivity and chemical stability with suitable inorganic lithium salts. Moreover, this material can be handled and processed easily, is low cost with good acid resistance, and is readily dissolved in solvents.

Anamika studied solid-state complementary ECD by assembling cathodic WO_3_ thin film and anodic NiO thin film on each side of the transparent ITO electrodes with a device structure of ITO/WO_3_/LiClO_4_-PC-PMMA-ACN/NiO/ITO. WO_3_ thin film was grown via the hydrothermal method while NiO thin film was electrodeposited on the ITO substrate. Initial stages of SSE preparation involved dissolving PMMA and LiClO_4_ in acetonitrile (ACN) to form a casting solution with a weight ratio of 3:7:20:70 (LiClO_4_:PMMA:PC:ACN). This solution was dried at 90 °C and used for ECD assembly. This ECD demonstrated fast colouring and bleaching times of 1.2 s and 1.5 s, respectively, with an optical modulation of 50.3% at 630 nm wavelength. In addition, the ECD showed a CE of 243 cm^2^ C^−1^, with an intercalated charge of 17.2 mC across an 8 cm^2^ surface area. Repeatedly cycling the device 20,000 times eventually increased the colouring time to 3.1 s and bleaching time to 3.9 s, which is attributable to the variation in crystalline structure and chemical composition of WO_3_ and NiO films after 20,000 cycles [31]. 

In another work on complimentary ECD, Liu et al. assembled an ECD with an ITO/WO_3_/LiClO_4_-PC-PMMA/NiO/ITO structure. Both the WO_3_ and NiO thin films were deposited via magnetron sputtering. Solid electrolyte was synthesised by blending PMMA powder in LiClO_4_-PC solution at a ratio of 30 wt.% and dried until a gel was formed. Then the electrolyte was applied over WO_3_ and NiO films for ECD incorporation. The ECD exhibited a colouring transmittance of 26.1% and bleaching transmittance of 77.8%, which resulted in a large optical modulation of 51.7% at 550 nm wavelength. Initial CA measurements revealed 4.5 s and 1.7 s were needed for colouring and bleaching of the device, respectively. After 1000 cycles, colouring and bleaching time extended to 5.0 s and 2.9 s, respectively. This is associated with the change in crystalline structure and composition of NiO and WO_3_ film after long cycling periods. In addition, a decrease of 16.8% in coloured transmittance during a 6 h period upon electricity disconnection demonstrated the memory effect of the complementary ECD, which is due to current leakage [32]. 

Lee et al. reported an all-solid-state ECD based on a simple and efficient approach in solid electrolyte fabrication for smart window applications. Methyl methacrylate (MMA) and LiClO_4_ salt were first dissolved in propylene carbonate (PC) purged by N_2_ gas. Subsequently, phenothiazine was added to the mixture and allowed to stir for a 12 h period at 60 °C. ECD was assembled by stacking ITO layers and sputtered WO_3_ layer with a 60 µm-thick Surlyn adhesive film, making up a working area of 2.34 cm^2^. Electrolyte was injected by drilling inlets on the ITO glass, and the ECD was subjected to UV curation. It is worth noting that this study suggested that prolonged UV curing would deteriorate the ECD performance due to immobile PMMA molecules blocking reaction sites at both electrode surfaces. The PMMA-based ECD cured at 10 min demonstrated exceptional device performance, with a large optical modulation of 51.3% recorded at 550 nm wavelength. In terms of switching characteristics, the time taken to colour the device was 1.5 s, while the decolouration process took 2.0 s. Additionally, the ECDs in this study showed outstanding stability of 98.9% after 11,500 cycles [33]. 

In another work, Evecan and Zayim constructed an all-solid-state ECD (ITO/WO_3_/LiClO_4_-PC-PMMA/ITO) for energy-saving systems. LiClO_4_ was first dissolved in can, and then PMMA was plasticised by the addition of PC for the formation of a highly transparent and conductive gel. Cyclic voltammetry (CV) measurements showed typical redox reaction of the WO_3_ working electrode when a voltage range of −3.2 V to 3.2 V was swept across. The time taken to achieve steady state was found to be approximately 10 s. In the CE study, the all-solid-state device exhibited 76.74% bleaching transmittance and 28.67% colouring transmittance at 630 nm wavelength, which led to a large optical modulation of 48.07%. Further analysis found that the CE was as high as 68.7 cm^2^/C [34]. 

Although PMMA and LiClO_4_ pairing is more commonly adopted, there exist reports on other Li salts as well. A combination of PMMA, LiBF_4_, 1-butyl-3-methylimidazolium trifluoromethanesulfonate (BMIMTOF) and dichloromethane (DCM) was prepared for solid-state electrolyte synthesis in the work of Lv et al. The all-solid-state polymeric ECD was assembled in such a way that poly(4,4′,4-tris [4-(2-bithienyl)phenyl]amine (PTBTPA) was deposited on ITO as a working electrode while poly(3,4-ethylenedioxythiophene):polystyrenesulfonate (PEDOT:PSS) films were deposited on another ITO as a counter electrode. The ECD was separated using a spacer and sealed with 3 M glue. Results revealed a fast colouring time of 1.02 s and bleaching time of 0.51 s under the influence of −1.4 V to 1.4 V. In terms of optical properties, a large optical modulation of 54.3% was recorded, which led to a large CE of 812 cm^2^/C. It maintained a good cycling stability of 70.2%, even after repeatedly cycled for 10,000 times [35].

### 1.2. Gelatine-Based SPE ECD

**Figure 3:**
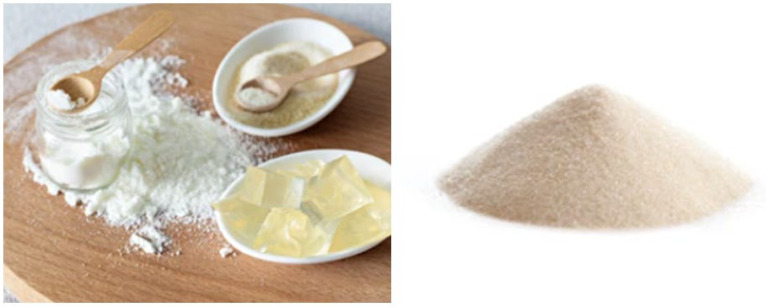
Commercially available gelatine powder for fabrication of SSE [45,46].

A bio-material that has caught the eyes of researchers, gelatine is a natural polymer derived from animal protein, often utilised for the synthesis of SSEs in ECD applications [36]. As shown in Figure 3, commercially available gelatine exists as powder and is a potential alternative for SSE studies because of its ability to generate electrolytes with huge viscosity, high transparency and high conductivity, not to mention it is a low-cost material and is abundant in nature [37]. In general, this material is frequently combined with formaldehyde and glycerol as a crosslinking agent and plasticiser, respectively, for enhanced membrane properties as well as adhesion to the electrodes [38].

Jatuphorn et al. investigated the performance of an ECD using gelatine-based electrolytes. Gelatine powder was mixed with glycerol before LiClO_4_ salt was added. The mixture was then blended continuously. Fabricated gelatine-based electrolyte film with 100–200 µm thickness was sandwiched between ITO/WO_3_ and ITO/NiO substrates to give rise to an ITO/WO_3_/gelatin-LiClO_4_/NiO/ITO ECD structure. In this work, the gelatine-based ECD was observed across an applied voltage of −3 V to 3 V while recording the results simultaneously. Findings on switching characteristics showed 10 s and 50 s, respectively, were needed to darken and lighten the ECD. Apart from that, it exhibited a colouring transmittance of 24.64% and bleaching transmittance of 71.02%, with an optical modulation of 43.35%. A CE of 53.90 cm^2^/C highlighted the competence of gelatine-based electrolytes in ECDs. Additionally, this work reported the addition of ethylene-acrylic acid ionomer further enhanced the EC performance. Although having a significant amount of its bleaching transmittance reduced (54.71%), a higher CE of 60.34 cm^2^/C was achieved, where diminished colouration charge played a crucial role [39].

In another similar work, Hossein and Jatuphorn reported the implementation of a gelatine-based LiClO_4_ solid electrolyte in an ECD with ITO/WO_3_/gelatin-LiClO_4_/ITO configuration. The gelatin-LiClO_4_ electrolyte was prepared by first dissolving gelatine in a cosolvent system of formic acid and acetic acid. Glycerol and LiClO_4_ were then added once complete dissolution was achieved in the previous step. Furthermore, this electrolyte possessed conductivity as high as 1.59 × 10^−4^ S/cm. CV results showed oxidation and reduction peaks, indicating good reversibility of the ECD. In the coloured state, it exhibited 43.8% transmittance, while in the bleached state it was 71.3%, with an optical modulation of 27.5%. CE was then calculated to be 69.37 cm^2^/C, which is higher than the reported CE (23 cm^2^/C) based on gelatine SSE in the work of Avellaneda et al. [40]. The large CE is mainly attributed to the large optical modulation with low colouration charge density (10.09 mC/cm^2^). The switching time was as quick as 1 s for the colwtouring and bleaching processes. On top of that, the authors had a different idea on constructing an ECD based on gelatine SSE. The WO_3_ working electrode was synthesised within the transparent adhesive gelatine SSE by the sol-gel method, which led to a ITO/WO_3_-gelatin-LiClO_4_/NiO/ITO device structure. Although demonstrating lower colouring transmittance (29.6%) and bleaching transmittance (54.4%), CE was still considerably high (51.54 cm^2^/C). The up side of this device structure is it only required about half the colouring charge (5.82 mC/cm^2^) compared to the earlier mentioned ECD. The authors explained that the lower colouring charge is due to the facilitation of Li^+^ ion transfer by incorporation of WO_3_ networks within the gelatine matrix. Colouring time was increased to 2 s, while bleaching time was 1 s [41].

In the work of Ramadan et al., they developed transparent gelatine-based solid electrolytes (GPSEs) for ECD application. Commercial grade colourless gelatine was boiled in in distilled water and stirred until complete dissolution. Next, formaldehyde was added as a cross-linking agent, acetic acid was added to prevent quick solidification of the electrolyte and glycerol was added as plasticiser. The introduction of different amounts of acetic acid (13–30 wt.%) under stirring completed the electrolyte fabrication process. As a result, a viscous solution was obtained and allowed to cool in a Petri dish for transparent membrane formation. On top of that, the highest conductivity of 1.28 × 10^−5^ S/cm was achieved at a 26 wt.% acetic acid as well as good mechanical stability and high transparency. With regards to ECD, the GPSE was sandwiched between a FTO/WO_3_ working electrode and NiO/FTO counter electrode. This ECD displayed an outstanding coloured transmittance as low as 1.9% and bleached transmittance of 69.6%, with a huge optical modulation of 67.7% at 600 nm wavelength under the effect of −2.5 V to 2.5 V. Subsequently, the CE was calculated to be 38.1 cm^2^/C. CA results revealed the response time to be around 60 s for both the colouring and bleaching process. On a side note, the authors mentioned hydrochloric acid is not a suitable conductive protonic contributor due to its corrosive nature, which deteriorates the quality of WO_3_ layer. The authors further explained that the linking between mechanical stability and optimised transmittance and conductivity is the contributing factor towards the efficiency of the ECD [42].

Apart from the conventional LiClO_4_ salt employed in SPEs for ECD applications, lithium iodide (LiI) salt is often employed as source of Li^+^ cations. Ponez et al. explored gelatine-based LiI/I_2_ as a possible candidate for ECD implementation. In addition, the authors reported an ionic conductivity of 9.8 × 10^−5^ S/cm at a LiI/I_2_ concentration of 15.7%. ITO was used as transparent electrode, WO_3_ was used as working electrode and Cerium dioxide–titanium dioxide (CeO_2_–TiO_2_) was used as ion storage layer. Subsequently, a small ITO/WO_3_/gelatin-LiI-I_2_/CeO_2_–TiO_2_/ITO was built with the mentioned layers. CV results showed typical curves after multiple cycles, which indicated the reversible nature of the device. As the device was being cycled repeatedly, its colouration time varied. At the 30th cycle, it took only 5 s to turn dark blue at −1.5 V. However, after 50 cycles, colouration time increased to 15 s at the same applied voltage. In contrast, decolouration required only 2 s. Furthermore, the ECD revealed an optical modulation of 20% as a result of a bleaching transmittance of 74% and colouring transmittance of 54% at wavelength 600 nm [43]. The reported findings for ECD here are similar to those listed by Heusing and Aegerter [44].

### 1.3. Ta_2_O_5_ Based SPE ECD

**Figure 4:**
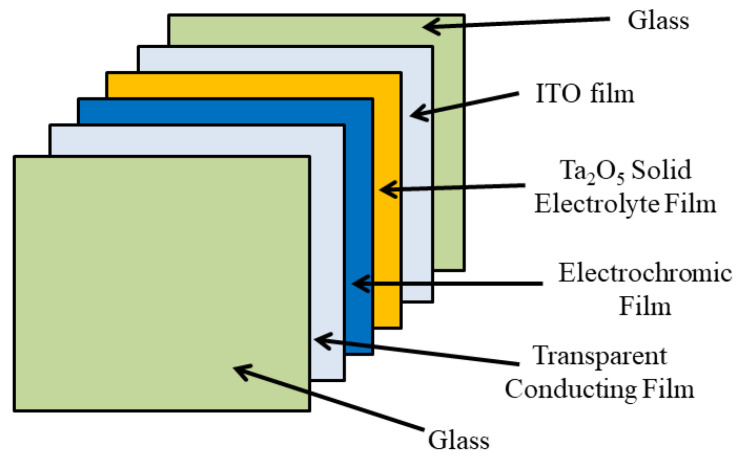
Structure of an all-solid-state ECD based on Ta_2_O_5_ solid electrolyte film.

Ta_2_O_5_ metal oxide films are known in the area of semiconductors for their application as dielectric layers, which includes storage capacitors in dynamic random access memories (DRAM), gate oxides in field effect transistors (FET) and anti-reflective layers for silicon solar cells [47,48,49]. Recently, this material has been studied as a proton-conducting solid electrolyte in ECDs to replace the traditional liquid electrolytes [50], as shown in Figure 4. This is attributable to its high transparency and its ability as an ion storage layer.

Chen et al. reported Ta_2_O_5_ films produced via the cathodic arc plasma method as solid electrolytes in an all-solid-state ECD. A 170 nm-thick ITO film on glass with low sheet resistance and high transparency was used as the deposition substrate. NiO film of 60 nm thickness was first deposited on the ITO substrate via DC pulsed sputtering. Ta_2_O_5_ film (130 nm) was subsequently deposited on the NiO/ITO/glass. At this stage, an electrolyte consisting of PC, LiClO_4_ and deionised water was injected into the NiO film by applying a constant voltage of −2.5 V. Eventually, the ECD fabrication was completed by depositing 160 nm-thick WO_3_ and 100 nm-thick ITO by magnetron sputtering. This gave rise to a ITO/WO_3_/Ta_2_O_5_/NiO/ITO device structure. The Ta_2_O_5_ layer grown at an oxygen to argon ratio of 2.4 appeared to exhibit a high ionic conductivity of 3.5 × 10^−6^ S/m, which is related to the porous and loose-columnar structure. Furthermore, CA results showed a large optical modulation of 50% in this ECD, with a considerably quick colouring time of 13 s and bleaching time of 5 s. The authors concluded that the fast response time was mainly due to the presence of voids in the Ta_2_O_5_ structure, which eased the ion transportation process. This was supported by the findings from electrochemical impedance spectroscopy (EIS), where a smaller semi-circle represents lower charge-transfer resistance [28].

An EC all-solid-state switchable mirror was developed by Tajima et al., which had a structure of Mg_4_Ni/Pd/Al/Ta_2_O_5_/H_x_WO_3_/ITO on glass substrate. Mg_4_Ni is the optical switching layer, Pd is the proton injecting layer, Al is a buffer layer, Ta_2_O_5_ is the solid electrolyte layer, H_x_WO_3_ is the ion storage layer and ITO is the transparent conducting layer. It is worth mentioning that H_x_WO_3_ is the outcome of sulphuric acid (H_2_SO_4_) injection across Ta_2_O_5_ film into the WO_3_ film at a constant voltage of 2 V. The device demonstrated a typical CV curve under a sweeping voltage, where it alternated between transparent and dark blue states. It is worth mentioning that after 100 cycles, the current density and charge insertion decreased due to the deterioration in film quality, which led to ion trapping. A total of 20 s was needed for device bleaching, while 10 s was needed for colouration. In addition, it changed from 10% in the coloured state to 48% in the bleached state. The authors further explained that in thinner Ta_2_O_5_ films, poorer optical properties manifested and took three times longer to alternate between coloured and bleached states [51].

An all-thin-film ECD featuring a reactively sputtered Ta_2_O_5_ solid electrolyte layer was reported by Rui Wang et al. Subsequently, it was incorporated into an ECD by stacking ITO, NiO, Ta_2_O_5_, WO_3_ and ITO on soda lime glass substrate by sputtering. As a result, an ECD structure of glass/ITO/NiO/Ta_2_O_5_/WO_3_/ITO was configured. It is worth mentioning that the authors claimed that working pressure played an important role in the functionality of the amorphous Ta_2_O_5_ solid electrolyte layers. Increased working pressure promoted a more porous structure, where transport of protons was made easier. In the best fabrication conditions, the more negative voltage during the colouration cycle was an indication of easier proton transport. In addition, this device showed good stability after repeated cycling for 780 rounds. The protonic conductivity was as high as 1.8 × 10^−6^ S/cm, as enough void spaces were provided for transporting channels for proton movement. Furthermore, the ECD exhibited ahigh optical modulation of 70% at 600 nm wavelength, with a fast colouring time of 17 s and fast bleaching time of 4 s. On the contrary, the other ECDs made at different working pressures exhibited much lower optical modulation and required longer switching times [52].

Li et al. investigated the effect of independently controllable electrolyte ion content on the performance of all-solid-state ECDs. A slightly different approach was adopted in this research, where Li metal of varying film thickness was used as electrolyte ions and introduced by resistive evaporation. The authors explained that in this approach, the Li^+^ concentration can be changed without altering the film thickness of the Ta_2_O_5_ layer. Various layers were stacked to construct an ITO/NiO/Ta_2_O_5_/Li/WO_3_/ITO, where each layer was deposited on each other accordingly to e-beam evaporation. It is noteworthy that the Li layer was not picked up by the SEM cross-sectional measurement, which indicated the diffusion of the mentioned layer into the other layers during the evaporation process [53]. In addition, 40 nm Li metal was found to be the optimum thickness, as thinner Li film showed deterioration in charge density and optical modulation, while thicker Li film led to large leakage current, low optical modulation and low CE. Initially, the ECD exhibited an optical modulation of 39.1%. However, it improved to as high as 53.7% after 500 cycles, which was attributed to the reduced Li ion transfer. Consequently, CE was observed to be 98.9 cm^2^/C, a slight improvement from its initial CE of 97.9 cm^2^/C. In terms of switching time, 62.5 s was observed for colouration while bleaching required 33.4 s. Although the switching characteristics were much slower than other reports, the approach in this work implied a great energy-saving effect in future ECD applications [54].

Apart from the preferred LiClO_4_ salt as ion contributor, Wang et al. attempted an all-solid-state ECD based on composite lithium titanate (LTO) and Ta_2_O_5_ via the magnetron sputtering method. The fabricated LTO films in this work were amorphous due to the ease of Li^+^ ion transportation. Along with the ability of Ta_2_O_5_ as ion storage layer, LTO film is capable of improving its ionic conductivity within its own amorphous structure. All the layers were stacked on one another to give the ITO/NiO/LTO/Ta_2_O_5_/WO_3_/ITO ECD structure. Moreover, the thickness of LTO and Ta_2_O_5_ was set at 280 nm and 1 µm, respectively. Results showed that high sputtering power led to the densification of film, which is a well-known cause for diminished performance in ECD. At the optimum sputtering condition of 100 W, the ECD exhibited a bleaching transmittance of approximately 65 % and colouring transmittance of approximately 1%. The authors claimed that a maximum of 68.3% optical modulation was achieved between 580–600 nm wavelength and an applied voltage of −3 V. It should be emphasised that low colouring transmittance is highly desired, as blocking incident sun rays is one of the main purposes in smart window applications. Additionally, the results revealed good memory effect characteristics in the ECD, where colouring transmittance was 12.8% after 3 days in the absence of applied voltage. This is an indication of good colouration depth and a way forward for energy saving devices. In the matter of response time, the all-solid-state ECD based on composite LTO/Ta_2_O_5_ film displayed 14 s and 13 s, respectively, for colouring and bleaching time at a sweeping voltage of ±3 V [55].

### 1.4. Nafion-Based SPE ECD

**Figure 5:**
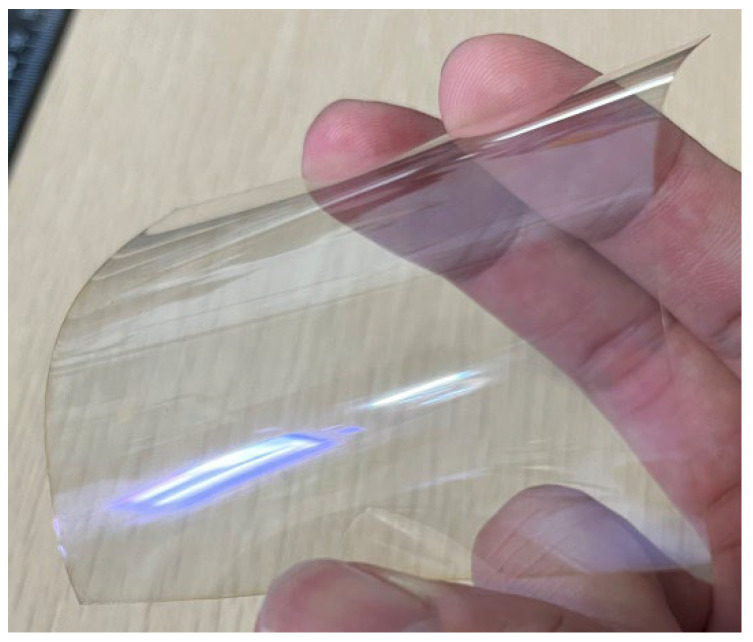
Transparent nafion polymer electrolyte membrane.

Nafion is one of the most common and commercially available polymer electrolyte membranes (PEMs) used in fuel cell applications, as shown in Figure 5. A product of Dupont, Nafion is a perfluorosulfonic acid polymer discovered in the late 1960s, consisting of a poly-tetrafluoroethylene (PTFE) backbone that supplies outstanding mechanical and chemical stability [56]. In addition, its distinctive proton-conduction ability is a result of perfluorinated side chains with a terminating sulfonic acid group. This is due to its fast cation transport and good thermal and electrochemical properties, which caught the attention of researchers to investigate its feasibility in ECD applications [57].

Cossari et al. attempted a simplified all-solid-state WO_3_-based ECD on a single substrate with the nafion chosen as SPE. WO_3_ films were fabricated via thermal evaporation, nafion SPE was fabricated via sol-gel spin-coating and ITO was deposited via RF sputtering. Each individual layer was stacked to form a glass/ITO/WO_3_/nafion/ITO (ECD-A) configuration. Additionally, another ECD was made with a slightly different device structure: glass/ITO/nafion/WO_3_/ITO (ECD-B). Morphological results showed the formation of a densely packed film structure with regular and homogeneous morphology in ECD-A. On the other hand, the WO_3_ film structure grown on top of nafion was observed to be less dense, with column-like microstructure that successively enabled smoother and more regularly sputtered ITO film. Consequently, difference in microstructure had an impact on the EC performance of ECD. ECD-B exhibited a colouring transmittance of 5% and bleaching transmittance of 75%, with an optical modulation of 70% at 600 nm wavelength. Compared to its ECD-A counterpart, this ECD too had a colouring transmittance of 5% but lower bleaching transmittance of only 55%. In addition, it took a large voltage of 12 V to achieve full colouration, which is not desired in ECD applications. In terms of switching kinetics, ECD-B was superior to ECD-A. A total of 90 s was needed for colouration in ECD-A compared to 5–10 s for ECD-B, which indicated the excellent charge transfer properties at the electrolyte/WO_3_ interface. In addition, ECD-A bleached within 30 s while ECD-B bleached in the range of 3–10 s. These results were further supported by EIS results, where ECD-B had the smallest semicircle in the Nyquist plot, indicating enhanced electron conductivity and proton diffusivity. In one of the most important aspects of the EC study, ECD-B exhibited a CE as high as 121 cm^2^/C. This high CE value was due to the large optical modulation coupled with a low colouration charge (6.82 mC) within the ECD [58].

In another nafion-based ECD report, Evecan et al. adopted a different approach for the fabrication of the WO_3_ layer, citing longer reaction time in the conventional methods. Ammonium tungsten oxide hydrate and oxalic acid were separately dissolved in distilled water before being combined into a single solution for further stirring. Subsequently, within a few seconds of the combustion reaction, as-synthesised powder was washed, filtered and dried, giving rise to solution combustion synthesis WO_3_ (SCS-WO_3_) film. The SCS-WO_3_ film had thickness of 150–200 nm and was uniformly coated. At the film level, it exhibited a typical CV curve in PC:LiClO_4_ solution under a sweeping voltage of −1 V to 1 V. Colouring and bleaching transmittances were observed to be 12% and 80%, respectively, with a remarkable optical modulation of 68%. Eventually, nafion electrolyte film was used as proton conductor in an ECD configuration of glass/ITO/SCS-WO_3_/nafion/ITO/glass. Under an applied voltage of −2.6 V to 2.6 V, the ECD demonstrated 86 % colouring transmittance and 23% bleaching transmittance. As a result, the optical modulation was 63% at 550 nm wavelength. Moreover, this ECD bleached within a few seconds and required approximately 30 s for colouration. A high CE value of 97 cm^2^/C was obtained, which was an indication of its comparability to existing reports on nafion electrolyte film ECDs [59].

Kattouf et al. reported mesostructured electrodes for fast switching all-solid-state ECDs by introducing nafion into the porous structure of the EC WO_3_ layer. As the proton reservoir, a 5 µm-thick nafion layer was deposited via the dip coating method. Preceding the construction of two ECDs, a dense WO_3_ and nafion-incorporated WO_3_ were subjected to proton charging in H_2_SO_4_ for 10 min in the standard three-electrode configuration. Subsequently, they were assembled into two ECDs, namely a bi-layer system (ITO/WO_3_/nafion/ITO) and hybrid mesostructured system (ITO/nafion-WO_3_/nafion/ITO), by using ITO-coated glasses as a counter electrode. At a sweeping voltage between −2.5 V and 1 V, the bi-layer system had 70% bleaching transmittance and a colouring transmittance of 35%. On the other hand, its hybrid mesostructured system counterpart had a higher bleaching transmittance of 80% and higher colouring transmittance of 45%. Both ECDs had a similar optical modulation of 35% at 632.8 nm. Moreover, the bi-layer system ECD took approximately 10 s for colouration, while the hybrid mesostructured system ECD took approximately 6 s for colouration. Despite the obvious difference in colouring time, their bleaching times of 2 s and 1.6 s were very close to each other. The authors further highlighted the main reason for the quicker time in hybrid mesostructured system ECD was due to the high interfacial area and contact, given its porous structure, that allowed more pathways for overall proton transfer [60].

Apart from the widely used WO_3_ in ECD structures, molybdenum oxide (MoO_3_) is one of the EC materials occasionally explored for its viability in ECD applications. Arash et al. demonstrated the use of nafion films in a MoO_3_ ECD, giving rise to ITO/MoO_3_/nafion/ITO. ITO-coated glasses were used as both ends of transparent conductors, MoO_3_ was deposited via physical vapour deposition and a nafion perfluorinated cation exchange membrane of 175 µm from Sigma Aldrich was used as solid electrolyte layer. This ECD showed high transparency during the bleaching state (80%) and low transparency during the colouring state (20%), resulting in an optical modulation of 60% at a 392 nm wavelength. Despite the large gap between colouring and bleaching transmittances, this was achieved by applying a large voltage of 7 V, where further increases in voltage indicated charged carrier saturation. Such saturation of charge was explained by the authors to be related to the MoO_3_ film thickness, further assuring that charge migration was hindered by the number of oxygen vacancies. In addition, response times of 8 s and 20 s were recorded for colouring and bleaching, respectively. Cyclic stability was only up to 7 cycles, where anything more had its optical modulation gradually decreased, attributing to the trapping of protons in the MoO_3_ structure. CE was subsequently computed to be as high as 116 cm^2^/C within an effective area of 4 cm^2^. It is interesting to note that the results obtained in this work were based on UV-A wavelength, which is different from the standard study in the near infrared region. Nonetheless, the authors did mention that the optical modulation of this ECD was 21.7% and the CE was 25.7 cm^2^/C at 630 nm [61].

A comparison of overall ECD performance adopting various solid electrolytes in the last decade is presented in Table 2 below. The solid electrolytes show encouraging results, which paves the way for more enhanced ECDs in the near future.

### 1.5. Post-Fabrication

Device sealing or encapsulation is equally important in the realisation of a complete ECD, as it protects the device from being exposed to the outer environment. Although this is not studied extensively, it is speculated that improper sealing may affect the physical structure of the ECD and subsequently its overall performance [62]. One of the most common methods is by applying commercially ready epoxy resin or silicone to the edges of the ECD [63,64,65]. This is a simple and direct method, where once the sealing of edges is completed, it is allowed to dry over a period of time to prevent moisture and oxygen from entering. In the work of Kao et al., device sealing was done by one layer of DuPont 60 µm Surlyn ionomer resin, which also acted as a reservoir for the electrolyte layer. It was first heated and pressed to seal the device. Eventually, the electrolyte was injected into the device through a drilled hole and cured for 40 min [66]. Other methods include hermetically sealing with Masterbond epoxy adhesive [67], UV curable epoxies [62] and polyisobutylene (PIB) [68], with an exclusive objective of keeping the inner structures of ECDs away from outer environment.

In the past decades, a great deal of emphasis has been placed on the durability study of ECDs. As the technology matures, it is essential and realistic that these devices are required to be able to function for a long duration [69]. Cycling durability is the foremost aspect, due to the fact that repeatedly switching on and off is a requisite for ECDs to have a long lifespan. In one study, Wen et al. reported galvanostatic rejuvenation of WO_3_ films by applying a constant current density of 10^−5^ Acm^−2^ through the sample for a period of 20 h. This was done to de-trap ions that are trapped in isolated deep sites, which subsequently decreased the charge density within WO_3_ films. The initial stage saw the potential rise from 2.8 V to 5.5 V. Once galvanostatic loading was done, the open circuit potential dropped to 3.3 V. The WO_3_ films were observed to have their original properties recovered [70]. There are numerous reports on this method in the literature [71,72,73].

Large area implementation has always been one of the biggest challenges in ECD study. There are several challenges that need to be addressed in order for the implementation of large area ECDs. As the contact area increases, an increase in sheet resistance will undoubtedly have an impact on the response time due to the fact that longer travelling distances between electrodes is required for conductive charges as well as the non-uniform distribution of the electric field [74,75]. Not only does the ECD show slow switching time, issues concerning the uniformity of optical contrast across the entire contact area may arise. As the primary objective of ECDs is to be implemented into smart windows for energy-saving purposes, high production cost is a stumbling block, which is bound to the nature of the fabrication technique and required materials. One way to compensate for this shortcoming is to extend the life cycle of ECDs to greater than 20 years. Despite the mentioned proposal, thermal stability of ECDs under real time conditions is still unclear. Given the multiple layers in an ECD, each individual layers has a different expansion coefficient, which affects the device mechanically and physically as it expands and contracts [76]. Consequently, the overall EC properties are affected.

### 1.6. Commercially Available Smart Glass

SageGlass is currently the most pioneering and advanced EC manufacturer in the world. A company founded about three decades ago, starting from reinventing the meaning of glass in buildings, it soon became the first to develop EC technology. In 2010, the world’s most energy efficient triple-pane glazing product was introduced to the EC market. Subsequently, the company was acquired by Saint-Gobain in 2012, a Paris-based world leader in building material for 350 years. They cover a wide range of dynamic glasses varying in size and colour. Additionally, sizes can be as large as 3 m × 1.5 m, while colours range from clear, to blue, green and grey. SageGlass Clear is one of smart glass products on the market. It has four levels of tint, namely clear, intermediate 1, intermediate 2 and full tint. In the clear state, it exhibits a transmittance of 60%, 18% transmittance in intermediate 1 state, 6% transmittance in intermediate 2 state and 1% in the fully tinted state. Moreover, it has a good solar heat gain coefficient (SHGC), ranging from 0.41 to 0.09, which specifies its ability to restrict heat energy from entering premises. This smart glass only requires 5 V to operate, and it takes approximately 7–12 min to switch between transparent and bleach states, depending ambient temperature and glass size. On average, it has a durability of 100,000 cycles or 30 years, operating between 30 °C and 60 °C [77].

View is another leading glass manufacturer, based in the US, that produces EC smart glass. Founded in 2007, the company was first named “eChromics” and renamed “Soladigm” later in the same year before changing its name to “View, Inc.” in 2012. View’s aim is to build smart windows in order to improve the health and well-being of people while at the same time saving energy. Similar to SageGlass, View has completed a great quantity of projects in buildings such as universities, hospitals and office buildings. Smart windows are installed in the mentioned places to provide a comfortable environment for occupants. As a result, employees in environments with optimised daylight and views reported 63% fewer headaches, 56% less drowsiness and 51% reduced eyestrain. Glare is the most significant cause for headache, related to the workplace lighting conditions. In addition, it is important to have a balanced circadian rhythm by having adequate exposure to natural light during the day. A lack of blue light exposure during the daytime has shown to lead to an uneven circadian rhythm, which lowers sleep quality at night and leads to drowsiness and fatigue during the day. While smart windows allow employees to work happier and healthier, optimising light exposure in buildings improves productivity and motivation as well [78].

Gentex Corporation is a world-renowned EC glass manufacturer for the automobile and aerospace industry. The company was founded in 1974 and is currently based in Michigan. Their mirrors use the principle of electrochromism to limit incident light from the headlamps of following vehicles. These mirrors are equipped with light sensors, proprietary gels and microprocessors to detect glare, thereby adjusting transparency accordingly while maintaining a clear vision for automobile users. This is advantageous. as it prevents night-time blindness, improves reaction time, decreases stopping distance and prevents driver fatigue. Besides exterior and interior mirrors, there are dimmable sunroofs, which darken on demand. In the transparent mode, its transmittance is more than 55%, while in the opaque mode, it can be as low as 1% in the visible light range. Moreover, it blocks up to 88% of total solar energy in the opaque state. Most notably, the Gentex smart glass is being used as windows in the Boeing 787 Dreamliner aircraft. It allows passengers to choose the desired level of darkness of the window, while enabling them to view the scenery and also lessen the dependence on air-conditioning systems.

## 2. Summary and Outlook

Electrochromism is a promising and important technology for energy-saving applications to address the increasing energy consumption in the modern era. In particular, smart glasses are specially designed to alter their transmittance according to external conditions. Furthermore, limited glare and heat are allowed to enter a building, thereby preserving comfortable indoor conditions while saving electricity costs. In recent years, significant progress has been made by researchers in the study of SSEs as an alternative to the existing LPEs, as a mean to address persisting leakage and evaporation issues, which may have an adverse effect towards the environment. This review presented some of the recent groundwork on PMMA, nafion, Ta_2_O_5_ and gelatine as solid electrolytes integrated in a multi-layered ECD prototype. It should be pointed out that these solid-state ECDs had remarkable performance. This provides insight for the next generation of smart glasses with enhanced mechanical and electrochemical properties. In addition, careful consideration must be given to the choice of materials, due to differences in material properties for the purpose of optimising a device.

Currently, costing remains a key factor in penetrating the smart window market. The cost of EC smart glass is estimated to be in the range of USD 50 to USD 100 per square foot by the National Renewable Energy Laboratory, which has proven to be a stumbling block in the spread of this technology. It has been further revealed that EC smart glass will begin to attract interest in commercial buildings if costing can be lowered to USD 20 per square foot [79]. However, minimising the costing risk compromises the overall quality of EC smart glass owing to the costing of raw materials and fabrication methods. As a result, continual efforts are necessary to work on low-cost multifunctional smart glasses while keeping maintaining their full potential. We believe that electrochromism is the way forward towards a greener and sustainable world while simultaneously reducing energy consumption on a daily basis. As our world becomes increasingly digitalised, these EC smart glasses can work hand in hand with the concept of the Internet of Things, where remote control is possible via mobile phones.

## Figures and Tables

**Table 1:** List of solid-state electrolyte candidates for ECD application.

Types of Solid Electrolyte	Description
PMMA	High-transparency thermoplastic material used for the formation of solid electrolyte.
Gelatine	Natural polymer derived from animal protein used for the formation of solid electrolyte.
Ta_2_O_5_	High-transparency metal oxide proton-conducting solid electrolyte film.
Nafion	Commercially available polymer electrolyte membrane.

**Table 2:** Summary of ECD performance using various solid electrolytes.

Author	Solid Electrolyte	Bleach Time (s)	Colour Time (s)	Bleach Transmittance (%)	Colour Transmittance (%)	Optical Modulation (%)	CE (cm^2^/C)
Anamika (2020)	PC-LiClO_4_-PMMA	1.2	1.5	–	–		243.0
Liu et al., (2016)	PC-LiClO_4_-PMMA	1.7	4.5	77.8	26.1	51.7	–
Lee et al., (2020)	PC-LiClO_4_-PMMA	2.0	1.5	–	–	51.3	–
Evecan and Zayim (2019)	PC-LiClO_4_-PMMA	10	10	76.7	28.6	48.1	68.7
Lv et al., (2020)	PMMA-LiBF_4_-BMIMTOF	0.5	1.0	–	–	54.3	812
Jatuphorn et al., (2020)	LiClO_4_-gelatin	71.0	24.6	–	–	43.4	54.9
Azarian (2020)	LiClO_4_-gelatin	1.0	1.0	71.3	43.8	27.5	69.4
Ramadan et al., (2017)	LiClO_4_-gelatin	60.0	60.0	1.9	69.6	67.7	38.1
Ponez et al., (2012)	LiI-gelatine		5.0	74.0	54.0	20.0	–
Chen et al. (2018)	Ta_2_O_5_	5.0	13.0	–	–	–	–
Tajima et al., (2011)	Ta_2_O_5_	20.0	10.0	48.0	10.0	–	–
Rui Wang et al., (2021)	Ta_2_O_5_	4.0	17.0	–	–	70.0	–
Li et al., (2020)	Ta_2_O_5_	33.4	62.5	–	–	53.7	98.9
Wang et al., (2018)	Ta_2_O_5_	13.0	14.0	65.0	1.0	68.3	–
Cossari et al., (2020)	Nafion	3.0–10.0	90.0	75.0	5.0	70.0	121.0
Evecan et al., (2019)	Nafion	–	–	86.0	23.0	63.0	97.0
Kattouf et al., (2013)	Nafion	1.6	6.0	80.0	45.0	35.0	–
Arash et al., (2020)	Nafion	20.0	8.0	80.0	20.0	60.0	116.0

## Data Availability

Not applicable.
